# Timely Catharsis

**Published:** 1883-08

**Authors:** Thomas N. Reynolds

**Affiliations:** Professor of Materia Medica and Therapeutics, and of Clinical Medicine in Detroit Medical College


### ARTICLE V.

TIMELY CATHARSIS. By THOMAS N. REYNOLDS, M.D., Professor of Materia Medica and Therapeutics, and of Clinical Medicine in Detroit Medical College. (Read Before the Michigan State Medical Society, and printed in this Journal with the Consent of the Society.)

Cathartics are as old as the history of man. They were used in every age, and the most primitive people observed their utility.

Native fruits, barks, and juices of plants are still used by the American Indians, and crude people of other countries. They use them with remarkably good judgment, but do not largely understand their *modus operandi* or their varied applicability. That wider knowledge and fine appreciation of the *modus operandi*, and consequent proper use of these remedies, as of those of every class, remains not only for a higher civilization, but for its more scientific and well trained physicians.

Generally it is not difficult to know whether to give or withhold some form of cathartic, but sometimes it requires of the most experienced the best consideration. A severe cathartic many times may be fatal; and so may withholding it be at others. In other cases, recovery may be retarded by too frequent purging, and in more, retarded by withholding the appropriate cathartic.

We will refer a little here to the use, rather than the abuse, of these remedies.

1st. In habitual constipation, where diet, exercise, regular habits, nervous tranquility and other advisory measures fail, it is necessary to give some laxative cathartic. The strength of the dose will vary with almost every individual; but the mildest should be used, if sufficient to produce one daily action of the bowels. Sometimes a glassful of warm water before breakfast will suffice; but, again, it may be necessary to begin with a large dose of some active cathartic, and then gradually decrease it to nothing at all. Generally this can be done ; but sometimes cathartics cannot be wholly dispensed with for any great length of time. Then some sufficient cathartic should be taken as required ; for though habitual constipation does not kill people suddenly, it does often kill people slowly. It is the important office of the lower bowel to remove from the body the unappropriated part of the food, and a large part of the excrementitious tissue waste. This failing, the kidneys are vicariously overworked, and from this undue irritation sometimes ultimately succumb to chronic interstitial inflammation. Every other organ and tissue also suffers from the presence in the blood of the reabsorbed excrementitious matter.

With regard to the kind of cathartic. Many think some one in particular is especially superior to every other, but that is not usually the fact. Any one of the scores of cathartics, taken in just sufficient quantity to produce the daily action, will generally accomplish the result as well as any other, provided the patient be not impressed with the paramount importance of having it do so at about a certain time. If this importance does become uppermost in the mind of the patient at a certain time of day, he will generally soon be able to live without any stimulus to the bowels but that of the mind, directing the necessary nervous energy to the performance of that function.

With most persons the morning is the best time. One of the excito-motor neurotic remedies is sufficient for some persons at times. Minims ii or iii of tincture of nux vomica in water, taken every hour for a few doses on rising, or the same of belladonna, will often suffice ; and warm coffee is a very constant and often quite prompt stimulus to intestinal peristalsis. Enemas may very properly be used in some cases, but continued enemas are not generally an advisable mode in chronic constipation.

2nd. Many cases of very extreme dyspepsia, that resist all other forms of treatment, and are found to have been preceded and accompanied by constipation, are relieved at once, and ultimately cured by gentle catharsis.

Magnesii sulphas is often the best selection. I sometimes prescribe:

℞ Magnesii sulph..............................℥i

Acidi sulphur. dil............................ʒij

Aquæ ad......................................℥iv

Misce et Sig.—Teaspoonful in water before eating.

3rd. In almost every injury or acute disease not connected with the bowel itself, and involving a taking to bed, an immediate evacuation of the bowels is a leading essential, if an existing laxness be not ascertained. It not only relieves the alimentary canal, but wonderfully relieves that high arterial tension, seen in most subjects immediately after the injury or reaction from shock, or after the invasion of any acute inflammation or essential fever. It acts as a revulsant of nervous energy from the circulatory apparatus, and thus lessens the abnormal frequency and violence of cardiac and arterial contractions. It may not always be best to repeat it if high arterial tension remains or returns, and it may sometimes be better to give hourly minim doses of tincture of aconite, or veratrum viride, or large doses of quinine; but many will bear a second cathartic action well, and will be more permanently benefited thereby.

4th. In acute lobar pneumonia of the plethoric, a prompt and active cathartic is very important, when given early. The benefit is threefold: It acts as an ordinary evacuant, depletes the general circulation, and diverts in a degree local vasomotor nervous excitement from the pulmonary vessels. Cathartics are useful in the same stage and same class of cases of acute lobar pneumonia that so many of the older practitioners found benefited by the abstraction of blood.

5th. In acute congestion of the liver from dietary causes, whether temporary and mild or more prolonged and severe, nothing is so radically beneficial as an occasional cathartic. Strict limitation of the dietary to cool acid drinks for the first few days is very largely curative in itself; but if the pulse be full and bounding at the wrist, a sharp purgative will act like a charm. I saw this marked in a young man lately, with hepatic congestion and an extreme supraorbital neuralgia. Of course quinine is indispensable in conjunction, if from malarial origin.

6th. In a case of trifacial neuralgia with severe and frequent spasmodic paroxysms, under my care at St. Mary's Hospital last winter, an active cathartic arrested the attack for forty-eight hours. The colon was full, however, and she had been on opium since the onset, three days before. Its return was not so severe, and she recovered slowly. Occasional anodynes sufficed when solid food was withheld. Solid particles excited a paroxysm when in contact with the palate.

7th. In acute rheumatism, particularly in robust subjects, an active cathartic in the early stage is wonderfully ameliorating to all the symptoms. In one young gentleman, the frequent subject of acute rheumatism, an active cathartic has, in a few instances, aborted the attack.

8th. In almost all acute head, or central, or cerebro-spinal symptoms, a brisk cathartic is likely to b every beneficial, acting in these mostly as a revulsant of innervation.

In active delirium from cranial injury, I have been much impressed with the benefit of an active cathartic. In January, 1874, George N., aged 14 years, received a fracture of the temporal bone, near the base, from the newly sharpened caulk of the shoe of a horse he was leading from the shop. His delirium was violent, and it was difficult to sew up his ear, which was cut nearly off by the same caulk that produced the fracture. It was impossible to give him a pill or anything but water. Since he drank with avidity, I conceived the idea of placing two drops of croton oil on water in a small glass. This he grasped at and drank, and in an hour and a half had several watery passages from the bowels. His delirium began to leave when his bowels began to move, and had disappeared when they ceased. It never returned, but his intellect was dull for some time after he got up. He forgot the names of things, and asked for beets as "some of those red things," at the table one day. Since this case, I have never forgotten the ease of giving a cathartic in the shape of croton oil in water, in active delirium, where patients drink with avidity, but become violent when desired to take anything else.

9th. In puerperal mania and puerperal eclampsia, a pronounced cathartic is important, and must rarely be contraindicated at the onset. In a plethoric woman it is indispensable if constipation exist, and I believe that nothing can then take its place in efficiency of service.

On July 19, 1878, I was taken by Dr. Wm. McDonald, then practicing in Detroit, but now practicing in Boston, to see a case of puerperal mania in a rather robust primipara. It was two and a half days after delivery, and after two days of unremitting puerperal insanity of the maniacal form. She was being forcibly held, screamed almost incessantly, and dashed from her everything but occasional drink. There was no uterine inflammation, the bowels had not moved, and we gave her three drops of croton oil in her next glass of water. A full evacuation followed, with two or three watery passages afterward. She grew gradually quiet, soon went to sleep, and had not any more symptoms of puerperal insanity.

In violent hysteria the benefit of a cathartic is often as great.

In puerperal eclampsia, a prompt and drastic cathartic, like three drops of croton oil, repeated, if necessary, will often arrest the paroxysms and cure the patient, unless too comatose and weak from frequent repetition. It is almost equally effective whether the eclampsia be from uræmic toxæmia, or from reflex nervous excitement. It acts in both by diverting vascular and nervous excitement from the head to the great mucous surface of the alimentary canal. In uræmia, it eliminates urea as well; and unless there be diarrhœa, it is a crime, then, to delay it a moment.

In two almost hopeless cases of puerperal eclampsia, of which I had personal knowledge, my brother, Dr. Henry J. Reynolds, after resorting to chloroform, etc., gave in each three drops of croton oil every two hours till nine drops had been taken before catharsis began. Purging soon followed, however, and in both recovery took place. These cases were reported to the Northeastern District Medical Society in 1881, and published on page 145 of No. 4, vol. v of the *Detroit Lancet*, and abstracted on page 554 of No. 4, Vol. II. of the *Quarterly Epitome of American*

*Medicine and Surgery*, supplement to *Braithwaite's Retrospect*, of the same year.

10th. In affections of the kidneys and skin, to speak in a general way, the bowels should be carefully kept sufficiently active. In many affections of each it cannot, of course, cure, but in none can it do harm, and in some, especially of the skin, a judicious diet and action of the bowels will effectually manage the malady.

I have had under observation for two years a generously living gentleman, aged 74, the subject of chronic eczema rubrum, in whom a single failure of the daily action of the bowels produces invariably, an increase of redness and almost intolerable itching, which nothing locally, excepting hot water, even temporarily relieves, but which is effectually removed by a resort to cathartics.

---

### THE MORAL STANDPOINT OF PORRO'S OPERATION.

Porro's operation is largely practiced in Europe at the present time. As a preventive against pregnancy on account of small pelvis, it is immoral and illegal, Cæsarean section offering more success, and it deprives the women of their procreative power, which is punished in different countries by from two to ten years penitentiary. Cohabitation with castrated women is immoral according to the religious laws of Christians and Jews. There are but two causes which justify Porro's operation. 1. Degeneration of the soft parts which cannot be removed without causing certain death. 2. Total atresia of the vagina, which impedes the secretion of the lochia, and occlusion of the vagina by non-removable tumors.—*Correspond. Blatt f. Schweizer Aertze.*

---

DR. ROSWELL PARK begs leave to announce that he has accepted the chair of surgery in the medical department of the University of Buffalo, and will consequently remove to that city during the latter part of August.

